# Effects of ultrasound-induced stress on gut microbiota of mice

**DOI:** 10.14202/vetworld.2023.929-938

**Published:** 2023-05-07

**Authors:** Irina Chernukha, Ekaterina Vasilevskaya, Ksenia Klimina, Roman Yunes, Nadezhda Kupaeva, Galina Tolmacheva, Anastasiya Kibitkina, Valery Danilenko, Sergey Karabanov, Liliya Fedulova

**Affiliations:** 1 Department of Experimental Clinic and Research Laboratory for Bioactive Substances of Animal Origin, V.M. Gorbatov Federal Research Center for Food Systems, Moscow, Russia; 2 Department of Genetics of Microorganisms, Vavilov Institute of General Genetics, Russian Academy of Sciences, Moscow, Russia

**Keywords:** antioxidant system, bacterial metabolites, gut microbiota, ultrasound-induced stress

## Abstract

**Background and Aim:**

Prolonged stress causes deleterious effects on both the organism and its microbiota. In this study, we examined the effects of exposure to variable frequency ultrasound (US) on the gut microbiota-liver-brain axis of mice.

**Materials and Methods:**

This study was conducted on 20 mature clinically healthy sexually naive C57BL/6J male mice (42–45 days old). Group 1 (Normal) consisted of healthy intact mice (n = 10). Group 2 (Stress) consisted of mice subjected to US-induced stress (n = 10) for 20 days with alternating frequencies (20–45 kHz). Stool samples were collected on days 0, 10, and 20, and the corresponding DNA was later subjected to 16SrRNA sequencing. After mice were sacrificed on day 21, the leukocyte count, blood serum biochemical parameters, and liver and brain antioxidant status were measured. Behavioral testing was performed on days 17, 18, and 19.

**Results:**

Ultrasound lead to higher stress and anxiety levels; increase in creatinine by 8.29% and gamma-glutamyltransferase activity by 5 times, a decrease in alkaline phosphatase activity by 38.23%, increase of de Ritis coefficient by 21.34%; increased liver and brain superoxide dismutase level by 20.8% and 21.5%, respectively; the stress-related changes in the gut microbiota composition – *Bacteroidaceae* and *Firmicutes*.

**Conclusion:**

Subjecting mice to 20 days of US-induced stress leads to systemic disorders due to oxidative stress and a decrease in the diversity of the gut microbiota.

## Introduction

Our modern lifestyle is marked by heightened susceptibility to “psychological” or “emotional” stress. It is fair to say that one of the main anxiogenic and depressogenic factors of our time is how we react to bad news that cause uncertainty such as pandemics, armed conflicts, disasters, economic, and social instability. Stressful life events can cause a number of psychological and physiological changes, including activation of the hypothalamic-pituitary-adrenal axis and the sympathetic nervous system [1], which are collectively known as “psychological reactions to stress.” Stress also contributes significantly to the disruption of the delicate balance between oxidants and antioxidants: Adenosine triphosphate suppresses antioxidant systems, which leads to oxidative stress and irreversible changes in cellular compounds, including proteins, carbohydrates, and lipids [2]. Stress causes a range of physiological changes in mice: Increased fear learning, learned helplessness, fatigue, and decreased reward motivation [3], often affects eating behavior, manifested by hyper or hypophagia [4], and also causes changes in steroid hormone levels [5]. Stress primarily affects the gut microbiota. The latter normally serves as a good indicator of the macroorganism’s physiological state and how much it is influenced by various factors [6]. Members of the normal gut flora flourish inside the body in the form of microcolonies fixed to certain receptors, enclosed in biofilms, which play an important role in the interaction between the macroorganism and the environment [7]. There are an increasing number of studies that clearly demonstrate there is a relationship between gut bacterial diversity and mental diseases, cognition, etc. [8]. Considering the anatomical and functional similarities between the gut and the liver, the gut microbiota-liver-brain axis has received increased attention in recent years as one of the important players mediating the onset and development of many diseases [9].

Many aspects of humans’ reaction to stress can be imitated in rodent models. Although rodent models do not accurately reproduce the biochemical or the physiological parameters of the stress response and cannot fully mimic the natural course of human disorders [10], some types of stress, such as audiogenic stress (noise exposure) are easier to study in rodents than others, mainly because this type of stress has been well-characterized and reactions of individuals to it have been carefully studied and documented [11].

News that causes uncertainty triggering depression-like behavior can be mimicked in rodents by exposing them to variable frequency ultrasound (US) [12]. The impact of US with a frequency of 20–45 kHz imitates an information flow that carries a negative emotional load and forms a state of learned helplessness, which is interpreted in animals as a depressive-like state. This model was previously described in male rats and mice in reference [12] to detect behavioral aberrations and molecular depression-like changes and in male mice [13, 14] to detect antioxidant changes in the brain. However, aspects of the effects of US-induced stress on the gut-liver axis have not been studied previously.

This article is dedicated to studying the effect of exposure to US on the gut microbiota and oxidative processes in liver and brain of male mice.

## Materials and Methods

### Ethical approval

The authors indicate that the procedures followed in this study are in line with the standards outlined in Directive 2010/63/EU of the European Parliament and the European Union Council for Protection of Animals used for scientific purposes. Animal studies were carried out in accordance with the NC3Rs ARRIVE guidelines for *in vivo* experiments. The research was approved by the V.M. Gorbatov Federal Research Centre for Food Systems of the Russian Academy of Sciences bioethical committee (protocol #03/2019, dated April 15, 2019).

### Study period and location

The study was conducted from May to June 2019 at the V. M. Gorbatov Federal Research Centre for Food Systems of Russian Academy of Sciences, Moscow, Russia. The stool samples were studied from July to September 2019 at the Vavilov Institute of General Genetics, Russian Academy of Sciences, Moscow, Russia.

### Animals

This study was conducted on 20 mature clinically healthy sexually naive *C57BL/6J* male mice (42–45 days old), which were purchased from the Federal State Budgetary Institution of Science “Scientific Center for Biomedical Technologies of the Federal Medical and Biological Agency,” Andreevka Branch (Moscow Region, Solnechnogorsk District, Andreevka Settlement). The mice were acclimatized to the new facility in harmonious groups for 7 days before the experiment.

The mice were kept in T-III polycarbonate cages (Techniplast, Italy) lined with hygienic bedding (Lignocel BK 8-15/LIGNOCEL, J. Rettenmaier and Sohne GMBH + CO KG, Germany), in groups of five, throughout the during all experiment. The cages were changed once every 7 days (but not <3 days before behavioral testing). For environmental enrichment, nesting material (Lignogel Nesting small, J. Rettenmaier and Sohne GMBH + CO KG) was used. The cages were changed once every 7 days (but not <3 days before behavioral testing). The mice were weighed every 7 days using electronic scales (Ohaus, Adventurer Pro, USA), which offer a precision of 0.1 g.

The mice were fed *ad libitum*, a wholesome feed compound (Laboratorkorm, Russia). The amount of food remaining after each day, including food on the bottom of the cages or food spilled onto plastic sheets placed under each cage, was weighed. The amount of consumed feed was calculated as the weight of the retrieved feed (in grams) minus the amount of provided chow.

The mice were kept under controlled environmental conditions: Air temperature 21 ± 2°C and relative humidity 50–60%. Lighting in the experimental rooms was artificial (12 h/day, 12 h/night), and daylight was inverted.

### Ultrasound stress model

Groups of mice were formed 2 days before the start of the experiment using the method of paired analogs (body weight and orienting-exploratory behavior in the “open field” test Porsolt pre-test-animals that did not display immobility in 15 min were suppressed).

Group 1 (Normal) consisted of healthy intact mice (n = 10).

Group 2 (Stress) consisted of mice subjected to US-induced stress (n = 10) for 20 days.

Group 2 was exposed to US-induced stress on day 0 in a separate laboratory room. The US-induced stress consisted of constant delivery of unpredictably alternating frequencies of US in the ranges 20–25 kHz and 25–45 kHz, and an average intensity of 50 ± 5 dB in accordance to previous studies [12, 14, 15]. The even distribution of US radiation was controlled using a US detector (Testo, Germany).

The US device (MNPF Aleks, Russia) was hung 2 m above the cages of the experimental groups. The average horizontal distance between the cages was 2.5 m. The position of the cages, with respect to the stimulator, was changed weekly.

### Completion of the experiment and sampling

Mice were placed individually in a plastic handling tube for stool sampling on days 0, 10, and 20. Stool samples were collected from each mouse to individual Eppendorf and stored at temperature minus (80°C ± 2°C).

On the 21^th^ day, the mice were euthanized using carbon dioxide in a VetTech installation (VetTech Solutions Ltd., UK), as stipulated the directive 2010/63/EU issued by the European Parliament and the European Union Council for Protection of Animals used for scientific purposes. Blood was collected from the right atrium into tubes with Ethylenediaminetetraacetic Acid (Aquisel, Spain) for analysis of whole blood and plasma parameters. Plasma was obtained by centrifugation using a CM-6M centrifuge (ELMI, Latvia) for 8 min at 2260× *g*.

Brain, liver, and adrenal glands were weighed by the electronic balance (Acculab VI-CON, Canada) to determine the relative mass of internal organs. Brain and liver samples were taken to analyze for the antioxidant activity analysis. Samples were frozen and stored at temperature minus (−41°C ± 1°C).

### Behavioral tests

Behavioral tests were performed on days 17 (Open Field and New Cage tests); 18 (Porsolt test), and 19 (O-maze and Porsolt tests). Behavioral analyses were performed by a blinded experimenter.

The open field test was performed in a square-shaped apparatus 0 × 40 × 30 cm (Open-Science, Russia), in which the central area received 100 lux generated by lights attached to the ceiling above [16]. The surface of the central area was 25 × 25 cm. Each mouse was placed in the center of the open field for 5 min. Their behaviors, including time spent in the center area (seconds), and outside of the center area, were recorded on Nikon DT5600 Kit (Nikon, Japan) and automatically processed using the Enthovision XT 14 software (Noldus, USA) and RealTimer (OpenScience, Russia).

The New cage test was used to assess the effect of the emetogenic novelty factor on animals [17]. The mice were housed in a standard plastic cage equal in size to their home cage. The number of exploratory rearings was considered as an indicator of vertical exploratory motor activity: They were counted for the whole 5-min period.

The Porsolt Forced Swimming test was adapted to the experimental conditions [18]. A transparent cylinder (17 cm) filled with water (23°C) was used for the test. The latent period and duration of immobilization were recorded on Nikon and automatically processed using Enthovision XT 14 software (Noldus). The absence of any directed movements of the head and body of the mice were labeled “immobilization behavior.” The mice were kept in the water for 5 min.

To measure the level of anxiety in the mice, we used the “O-maze” test (OpenScience) [19]. The maze consists of a black 10-cm-wide acrylic path that goes in a circle 105 cm in diameter that is elevated 72 cm above the floor. The maze was divided into four quadrants of equal length with two opposite open quadrants with clear acrylic borders 1 cm high to prevent falls and two opposite closed quadrants with black acrylic walls 28 cm high. The total duration of the test was five minutes. The latency of the first exit and the total number of exits from the dark closed arms of the apparatus were considered as indicators of anxiety-like behavior.

### Blood samples analysis

The functional activity of leukocytes (relative content of lymphocytes, granulocytes, and monocytes) was assessed using a Guava easyCyte automatic flow cytometer (Merck Millipore, Germany).

The biochemical parameters of blood plasma; total protein, albumin, creatinine, urea, aspartate aminotransferase (AST), alanine transaminase (ALT), alkaline phosphatase (ALP), lactate dehydrogenase, gamma-glutamyltransferase (GGT), triglyceride, cholesterol, and glucose were measured using a BioChem FC-360 automatic biochemical analyzer (HTT, USA) using reagent kits (HTT). The de Ritis coefficient was calculated as the ratio of AST and ALT activity in relative units, according to Chernukha *et al*. [20].

### Brain and liver antioxidant activity

The liver and brain were homogenized vigorously in glass tissue homogenizer (LabForce, USA) with 50 мM phosphate buffer (pH = 7.0) in 1:10 ratio, and centrifuged at 5000 g for 5 min at 4°C in a centrifuge 5427R (Eppend-of AG, Germany). The supernatants of each sample were separated and stored at −20°C and used later for assessing the antioxidant properties.

The catalase (CAT) activity in organ extracts was determined using a spectrophotometer SF-2000 (OCB “Spectr”, Russia) following the method described by Chernukha *et al*. [21]. 720 μL of 50 mm phosphate buffer (pH = 7.0) was mixed with 800 μL of 0.1% hydrogen peroxide (PanReac AppliChem, Germany). The optical density (D0) was measured at a wavelength 240 nm relative to the control sample, using 1 cm wide cuvettes. Then, 20 μL of the brain extracts or 5 μL of liver extract were added to the test tubes. After 1.5 minutes of incubation, the optical density (D1) was measured at a wavelength of 240 nm. For the control samples, 800 μL of phosphate buffer was added instead of hydrogen peroxide. The activity was calculated using the equation:

U = (D0–D1) · r · 106/ε/t/Cpr (2) (1)

Where D0: the optical density before sample addition; D1: optical density after incubation; ε: coefficient of molar extinction of hydrogen peroxide (39.4 M^−1^ cm^−1^); t: incubation time (1.5 min); Cpr: protein concentration (g/L). The results were expressed as U/g of protein, in which U is the amount of H_2_O_2_ (mmol) neutralized per min.

The superoxide dismutase (SOD) activity in organ extracts was determined using a spectrophotometer SF-2000 (OCB “Spectr”), according to Chernukha *et al*. [21]. The volume of 1.14 mL of 50 mm phosphate buffer (pH = 8.2), 30 μL of extract and 30 μL of 10 mM pyrogallol solution (PanReac AppliChem) were added and mixed. The increase in the optical density of the experimental samples was measured at the beginning (D0) and after 2 min of incubation (D1) at a wavelength of 340 nm relative to the phosphate buffer, using 1 cm wide cuvettes. Auto-oxidation of pyrogallol was measured in a control sample in the same reaction mixture, adding 30 μL of 50 mM phosphate buffer instead of the sample. The percentage of inhibition of pyrogallol auto-oxidation was calculated using the equation (2 and 3) and SOD activity was calculated using the equation (3):

P% = (ΔDcon-ΔDex) · 100%/ΔDcon (2)

U = P%/t/Cpr/Vs (3)

In which ΔDcon: Difference in optical densities before incubation and after for the control sample; ΔDex: Difference in optical densities before incubation and after the test sample; t: incubation time (2 min); Cpr: protein concentration (g/L); Vs: sample volume (mL). The results were expressed in U/g of protein, in which U percentage of pyrogallol inhibition per min.

Protein was measured using the semiautomatic analyzer BioChem SA (High Technology Inc., Walpole, MA, USA) with the corresponding commercial kits (High Technology Inc.) following the manufacturer’s instructions.

### DNA extraction and 16Sr RNA sequencing

DNA was extracted using “Sorb-GMO-A” (Cat.N.GM-502-50, SYNTOL, Russia) following the manufacturer’s instructions. 16S library preparation and sequencing were carried out following Illumina’s protocol (16S Metagenomic Sequencing Library Preparation). Briefly, extracted DNA was amplified using standard 16S rRNA gene primers, complementary to the V3-V4 region and containing 5’-illumina adapter sequences. In the next step, individual amplicons were polymerase chain reaction-indexed and pooled. DNA libraries were sequenced on a MiSeq instrument (Illumina, San Diego, CA, USA) using Mis-eq reagent kit v2 (Illumina).

### Statistical analysis

Statistical processing of the results was carried out using the program “statistical package for the social sciences statistics 23.0” (IBM Corp., NY, USA). Intergroup comparison was performed using pairwise comparison of groups using the nonparametric Mann–Whitney test. The results are presented as a median (Me) and interquartile range ([P25–P75]). A p = 0.05 or less was considered statistically significant.

## Results

### Weight and food intake

**Figure-1:**
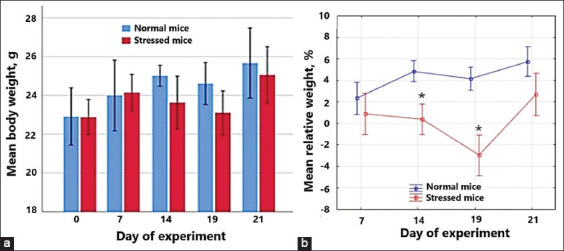
Mice body weight throughout the experiment (a) The results are presented as a median (Me) and interquartile range (P25–P75); mean body weight relative to day 0 (b) *is p < 0.05 compared to the mean of normal mice.

Normal mice gradually gained weight (Figure-1), reaching by the end of the experiment a 13.03 (7.38–16.64) g increase as compared to day 0 of the experiment. There were no significant differences in the patterns of body weight change over time among the groups. Normal mice showed no difference in food intake during the experiment; the average daily feed consumption ranged from 12 to 14 g/day per mouse. The stressed mice lost 1%–3% (p = 0.010) of their weight between days 14 and 19. On the 21^th^ day of the experiment, the mice gained up an average of 9.91 (8.58–12.13) g relative to day 0, which is explained by the weight loss that was observed in 30% of control mice by the end of the experiment. From day 1 to day 7, the average daily feed consumption ranged from 12 to 14 g/day per mouse in both the control and the intact groups. From day 7 to day 21, the amount of feed consumed dropped to 8–10 g/day per mouse.

### Behavioral tests

**Figure-2:**
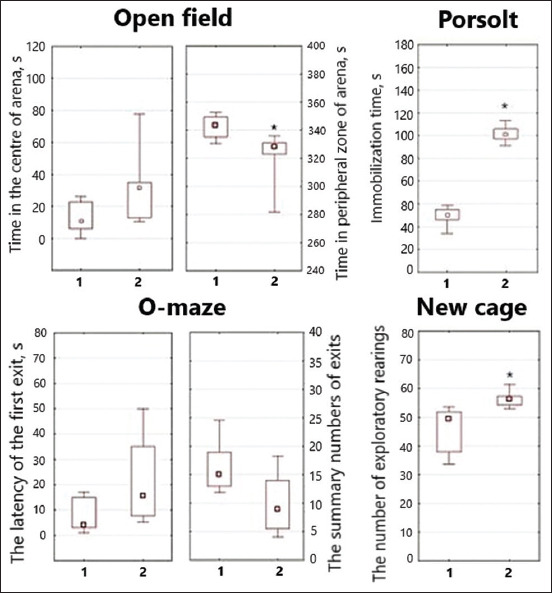
Summary of behavioral test results obtained on days 17–19 of the experiment. Data presented as box plots illustrate hinges extending from the 25^th^ to 75^th^ percentiles, the median line within the box and whiskers extending to the minimum and maximum values of the dataset. 1 – Normal mice; 2 – Stressed mice. * is p < 0.05 compared to the median of normal mice.

The results obtained in behavioral tests are presented on Figure-2.

Comparison of the stressed mice to the non-stressed mice in Open field test revealed a 57.14% (p > 0.05) increase in the duration of time spent in the center of the arena and a decrease of time in the peripheral zone by 5.80% (p = 0.048). In the Porsolt test, a 67.68% (p < 0.001) increase in the duration of immobilization was revealed. One possible explanation is reduced affect display (emotional blunting).

Moreover, in the O-maze test, a 4.25-fold increase in the latency period of the first exit (p > 0.05) and a decrease in the overall number of exits by 46.67% (p = 0.059) were observed. In the New cage test, the number of exploratory rearings increased by 8.0% (p = 0.024). It confirmed the increased anxiety of mice because of prolonged exposure to US.

### Results of blood and plasma analysis and antioxidant capacity

Blood cytometric parameters and plasma biochemical parameters are presented in Table-1.

**Table-1:** Blood and plasma changes of the experimental group on day 21 of the experiment. The results are presented as a median (Me) and interquartile range (P25–P75). * is p < 0.05 compared to the median of normal mice.

Parameter	Normal mice	Stressed mice
Whole blood parameters		
White blood cells, 10^9^	2.0 (1.3–5.9)	2.0 (1.8–4.1)
Lymphocytes, %	83.8 (80.1–84.8)	84.7 (83.1–85.7)
Granulocytes, %	13.1 (11.9–17.3)	11.5 (9.7–14.2)
Monocytes, %	3.1 (2.5–4.2)	3.4 (2.8–3.8)
Plasma biochemical parameters		
Total protein, g/L	42.6 (41.2–44.3)	42.7 (40.6–44.4)
Albumin, g/L	32.0 (31.0–33.0)	32.0 (30.0–32.0)
Creatinine, µmol	53.1 (49.8–55.8)	57.9 (56.1–58.2)*
Urea, mmol/L	10.2 (9.0–11.4)	9.5 (9.2–9.7)
AST, U/L	104.5 (99.8–135.0)	131.2 (88.5–141.0)
ALT, U/L	55.6 (51.1–64.4)	54.9 (52.5–59.1)
ALP, U/L	7.4 (5.9–8.2)	4.0 (0.3–4.7)*
GGT, U/L	0.1 (0.1–0.5)	0.5 (0.1–0.9)
LDG, U/L	262.1 (235.1–281.0)	255.2 (218.2–270.6)
Glucose, mmol/L	8.9 (7.5–10.1)	9.2 (8.3–9.7)
Cholesterol, mmol/L	2.9 (2.6–3.8)	2.8 (2.3–3.4)
TGr, mmol/L	1.3 (1.1–1.3)	1.1 (1.0–1.2)
De Ritis, RU	1.88 (1.69–1.95)	2.39 (2.10–2.48)*
CAT, U/g protein		
Brain	17.2 (15.6–18.3)	17.9 (12.9–27.0)
Liver	88.9 (52.6–97.3)	63.5 (56.8–63.8)
SOD, U/g protein		
Brain	209.4 (185.9–240.8)	254.5 (254.0–390.7)*
Liver	95.3 (95.0–95.7)	115.1 (104.6–126.6)*

AST=Aspartate aminotransferase, ALT=Alanine transaminase, ALP=Alkaline phosphatase, GGT=Gamma-glutamyltransferase, CAT=Catalase, LDG=Lactate dehydrogenase, SOD=Superoxide dismutase, RU=Relative units

According to our results, stress did not affect the white blood cell count in stressed mice.

We did not find any significant differences in levels of proteins (total protein, albumin, and urea) and lipid (cholesterol and triglycerides) related to metabolism, nor did we see any differences in glucose level.

However, we observed major changes in the liver biochemical markers of stressed mice: an 8.29% (p = 0.047) increase in creatinine levels, a 38.23% (p = 0.011) decrease in ALP activity, an increase in de Ritis coefficient by 21.34% (p = 0.039), and a five-fold increase in GGT activity (p = 0.491) as compared to normal mice.

The liver’s CAT activities decreased by 28.6% (p = 0.212) and SOD activity increased by 20.8% (p = 0.038), respectively, in the stressed mice as compared to the normal mice. Furthermore, there was increase SOD activity in brain tissues of stressed mice by 21.5% (p = 0.012).

### Internal organs’ relative weight

**Figure-3:**
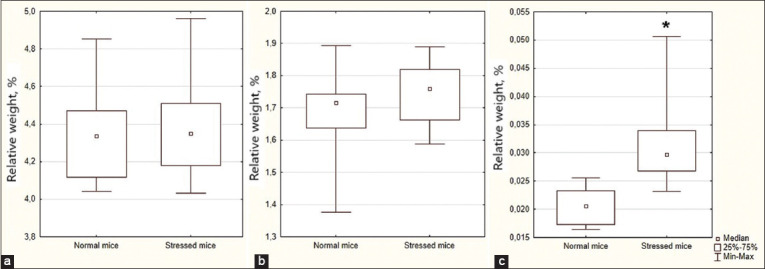
Relative weight of liver (a), brain (b), and adrenal glands (c) in Normal and Stressed mice. The results are presented as a median (Me) and interquartile range (P25–P75). * is p < 0.05 compared to the mean of normal mice.

We observed no differences in the relative weight (Figure-3) of the brain and the liver. However, we noted a 34.70% (p = 0.003) increase in the adrenal gland’s weight in stressed mice compared to normal mice.

### Mice gut microbiota

**Figure-4:**
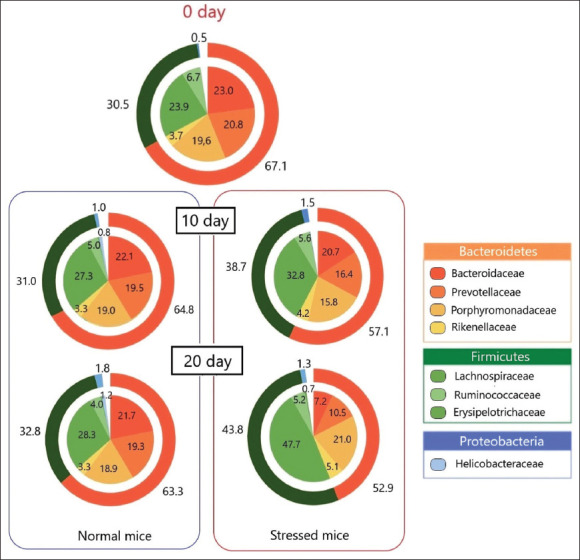
Changes in the gut microbiota composition over 20 days in Normal and Stressed mice. A diagram for 0 day is common for both groups.

On day 0 of the experiment, the gut microbiota of mice was mainly represented by the following phyla: *Bacteroidetes* (67.1%), *Firmicutes* (30.5%), and *Proteobacteria* (0.5%) (Figure-4).

On day 20, the gut microbiota composition of normal mice changed slightly: the abundance of *Bacteroidetes* decreased (63.3%), *Firmicutes* (32.8%), and *Proteobacteria* (1.8%) increased. The main changes were related to redistribution within the *Proteobacteria* families: the number of *Helicobacteraceae* (initially 0.23%) increased to 1.19%, *Sutterellaceae* (initially 0.16) – became 0.18; two new families emerged that were not detected on day 0 – *Desulfovibrionaceae* (0.21%) and *Enterobacteriaceae* (0.21%).

The impact of US stress led to a significant change in the composition of the gut microflora on the 10^th^ day as compared to the onset of the experiment: at the phylum level, the total number of *Firmicutes* rose to 38.7% (an increase of 25.8%, p = 0.042), which can be explained by an increase in bacteria of the *Lachnospiraceae* family (by 27.1%, p = 0.044) and the emergence of two families – *Erysipelotrichaceae* (0.15%) and *Lactobacillaceae* (0.11%, p = 0.036); and the emergence of *Verrucomicrobia* (0.29%, p < 0.001). Similarly, some important changes took place at the family level, namely, *Proteobacteria*. *Sutterellaceae* increased to 1.03% (more than six times, p < 0.001). The abundance of *Helicobacteraceae* (initially 0.23%) dropped almost two-fold (p = 0.031). Moreover, two new families, *Enterobacteriaceae* (0.21%) and *Desulfovibrionaceae* (0.14%), emerged.

On day 20 of the experiment, the changes observed on day 10 in the stressed mice developed further. We observed significant changes at the level of the phylum: *Bacteroidetes* dropped by 16.4% (p = 0.014), *Proteobacteria* dropped by 27.8% (p = 0.042), and *Firmicutes* increased by 25.0% (p < 0.001). Within the *Bacteroidetes* phylum, *Bacteroidaceae* and *Prevotellaceae* dropped by 2.7 times (p < 0.001) and 79.9% (p = 0.008), respectively, and the abundance of *Rikenellaceae* increased by 1.5 times (p = 0.039). Within the *Firmicutes* phylum, *Lachnospiraceae* and *Ruminococcaceae* increased by 170% (p < 0.001) and 22.9% (p = 0.046), respectively. As far as the *Proteobacteria* phylum is concerned, we observed a 42.0% (p = 0.023) decrease in the *Helicobacteraceae* family, and a 220% (p < 0.001) increase in *Sutterellaceae*. *Enterobacteriaceae* have disappeared altogether (p < 0.001).

We identified 28 genera in total in the gut microbiota of normal mice. The genera representing the majority (95%) were from more abundant to less abundant, *Bacteroides, Alloprevotella, Prevotella, Alistipes, Paraprevotella, Ruminococcus, Saccharibacteria genera incertae sedis, Parasutterella, Odoribacter*, and *Barnesiella*. Stress altered the composition of the gut microflora of mice significantly: *Ruminococcus, Parabacteroides*, and *Akkermansia, Lactobacillus, Dorea, Blautia* and *Rothia* disappeared. We observed a ≈ 50% (p = 0.029) decrease in *Alloprevotella* leading perhaps to their replacement by *Alistipes* (p = 0.035), *Saccharibacteria genera incertae sedis* (p = 0.022), *Rikenella* (p < 0.001) and *Odoribacter* (p = 0.006) occupying a free niche. We also observed a simultaneous decrease in bacteria of the genus *Bacteroides* and an increase in the abundance of *Clostridium* (*Clostridium XlVa*, p = 0.038; *Clostridium IV*, p = 0.021) and *Alistipes* (p = 0.016) were revealed.

Thus, we can conclude that stress caused a significant decrease in the microbial diversity of mice.

## Discussion

Our study supports the existing theory that audiogenic stress causes changes in mice comparable to informational stress in humans.

In our study, we reproduced a previously developed model of US stress in male mice [5, 12, 13, 15]. In the Open field test, the stressed mice spent 5.80% less time in the peripheral zone. The stressed mice also displayed a longer duration of immobilization in the Porsolt test, reaching 67.68%. In the new cage test, the number of exploratory rearings carried out by the stressed mice was 8.0% higher as compared to non-stress mice. Behavioral testing of mice exposed to US-induced stress confirmed their increased anxiety, emotional blunting, and depressive-like behavior, which is in line with previously published studies [5, 13, 15] and indicates that the mice developed chronic stress as a result of ultra-sound-induced negative information load [12]. This fact is confirmed by an increase in the relative weight of adrenal glands in stressed mice (by 34.70%).

Ultrasound-induced stress eventually leads to the development of systemic disorders. First example, brain-energy crisis was manifested in an increase of SOD (by 21.5%) activity aimed at preventing protein oxidation and lipid peroxidation in the brain [22–25]. Hepatic dysfunction was also manifested by an increase in the de Ritis coefficient (by 21.34%), creatinine (by 8.29%), and a decrease in ALP activity (by 38.23%) [26, 27]. SOD activity increased by 20.8%, most likely as a result of the increased production of superoxide anions [28]. It is noteworthy that the free radical scavenging activity of SOD is effective only when it is followed by the action of CAT. In this study, we observed the inhibitory effects of stress on CAT levels in the livers of stressed mice. This is explained by the fact that the dismutase activity of SOD generates hydrogen peroxide resulting from its interaction with the superoxide ion, which is more toxic than oxygen-derived free radicals and is further eliminated by CAT [29, 30].

As we expected, changes in the physiological functions and biochemical parameters were accompanied by changes in the abundance of gut microorganisms. Some of these bacteria could be considered as biomarkers of stress.

In our study, we observed a decrease in *Bacteroidaceae*, namely, in the genus *Bacteroides* and an increase in the abundance of the genus *Clostridium*, which is congruent with the data obtained on a heat stress rat model by Qu *et al*. [31]. We also observed an increase in the phylum *Firmicutes*. Zheng *et al*. [32] found an association between changes in the abundance of the *Bacteroidaceae* family (*Prevotellaceae*) and depression. In the same study, bipolar disorder was associated with altered levels in *Firmicutes* (*Lachnospiraceae* and *Ruminococcaceae*) [33]. An increase in *Lachnospiraceae* has also been reported in other stress-related studies [34, 35].

We also observed an increase in the abundance of *Odoribacter, Saccharibacteria genera incertae sedis*, *Rikenella*, and a decrease in *Alloprevotella*. In other studies dedicated to the effect of stress on the gut microbiome, the data on these genera of bacteria are not consistent.

For example, Chi *et al*. [7] reported a stress-related decrease in *Odoribacter* and *Rikenella* in mice. However, Li *et al*. [36] observed an increase in *Odoribacter* in a mouse model of depression. Bai *et al*. [37] demonstrated that *Alloprevotella* were characteristic of chronically stressed mice. We did not find any studies in which the presence or absence of *Saccharibacteria* was correlated with stress in mice or other laboratory animals.

A recent study conducted by Kraimia *et al*. [38] found that chronic stress leads to an increase in bacteria of the genus *Alistipes* in quails, which is in line with other studies [35, 39]. According to Kraimia *et al*. [38], the negative impact of bacteria of the genus *Alistipes* can be explained by the production of pro-inflammatory lipopolysaccharides that increase intestinal permeability and enter the bloodstream, which leads to neuroinflammation and behavioral changes [38, 40]. These data are well correlated with behavioral results.

To see how our results showing how US stress in mice affects their gut microbiota would overlap with human studies, we scrutinized the literature focusing on the influence of information stress on the gut microbiota of humans.

In the previous studies [41–43], the authors reported a decrease in *Bacteroidaceae* and *Prevotellaceae* in patients with depression, which is in line with our results. Overall, there is still some debate on how exactly *Lachnospiraceae* and *Ruminococcaceae* are related to depression.

The changes in the diversity of the gut microbiota that we observed are associated with the microbiota-liver axis. Changes in the liver’s functioning described above could affects directly host the flow of bile acids which are known to control microbial diversity. Similarly, microbial metabolism of these bile acids drives host physiology [44]. Such processes can alter the intestine’s pH, which creates conditions that are favorable to some bacterial genera and less favorable to others. The increased abundance of *Clostridium* in stressed mice suggests that one of the possible physiological manifestations of stress may be an increase in the concentration of bile acids. *Clostridium* are known to be some of the most sensitive bacteria to bile acids, mainly because they convert cholic and chenodeoxycholic acids (major bile acids) into metabolites that increase pH [45, 46]. However, more research is required to confirm this link [47].

To summarize, exposing mice to US influenced the gut microbiota-liver-brain axis significantly leading to (1) A decrease in the diversity of the gut microbiota (rearrangement of families and genera); (2) changes in liver function (lipid peroxidation and, probably, a change in the profile of bile acids); and (3) a brain-energy crisis (a change in behavior and a disruption of the prooxidant-oxidative balance).

It is crucial to be able to follow-up on this study to identify markers of serotonin and serotonin precursors in the brain and caecum contents, and to study the effects of US on cognitive functions. These studies will help us better understand the mechanisms of gut microbiota-liver-brain axis at the molecular and genetic levels.

Our results suggest that, similar to humans, stress takes a cumulative toll on the organisms of mice, leading to the development of a wide range of systemic damage [48–50].

## Conclusion

Based on the behavioral tests, biochemical profile, brain and liver antioxidant activity and stool samples 16Sr RNA sequencing of the tested mice, we propose that 20 days of US-induced stress lead to the development of systemic disorders due to oxidative stress and changes in the diversity of the gut microbiota. A decrease in *Bacteroidaceae* and *Prevotellaceae* increased abundance of *Firmicutes* and *Clostridium* that we observed are associated with the microbiota-liver axis. Further studies will help us better understand the mechanisms of gut microbiota-liver-brain axis at the molecular and genetic levels and should be investigated to enhance the health and welfare of animals and humans.

## Authors’ Contributions

IC and VD: Conceptualization, data curation, writing review, and editing. KK: Methodology. GT: Methodology, formal analysis, and investigation. RY: Formal analysis, investigation, and writing original draft preparation. AK, SK, and NK: Investigation. EV: Writing original draft preparation, visualization. LF: Writing original draft preparation, visualization, project administration, and funding acquisition. All authors have read, reviewed, and approved the final manuscript.
